# Synthesis of Mg-Al layered double hydroxides by electrocoagulation

**DOI:** 10.1016/j.mex.2018.07.019

**Published:** 2018-08-08

**Authors:** Marena Molano-Mendoza, Dayana Donneys-Victoria, Nilson Marriaga-Cabrales, Miguel Angel Mueses, Gianluca Li Puma, Fiderman Machuca-Martínez

**Affiliations:** a Escuela de Ingeniería Química, Universidad del Valle, A.A. 25360 Cali, Colombia; b Department of Chemical Engineering, Universidad de Cartagena, A.A. 1382, Postal 195, Cartagena, Colombia; c Department of Chemical Engineering, Loughborough University, Loughborough LE11 3TU, United Kingdom

**Keywords:** Synthesis of Mg-Al layered double hydroxides by electrocoagulation, Mg-Al-LDH, Gibbsite, Brucite, Waste water treatment, Ion removal

## Abstract

Recently, layered double hydroxides (LDHs) have attracted much consideration due to their versatility and easily manipulating properties and their potential applications such as anion exchangers, support of catalysts, flame retardants, biomedical drug delivery. A novel method for the *in-situ* preparation *in situ* of LDHs, using electrocoagulation (EC) processes was developed, the EC process was performed under two different conditions, at 5 mA m^−2^, changing polarity of the electrodes to find out the composition that leads to LDHs generation. The final product was characterized using XRD, BET and FTIR techniques.

This method presented the following advantages: (1) Simultaneously LDHs synthesis and wastewater treatment by ion removal; (2) Polarity control allows to manipulate the M^2+^/M^3+^ molar ratio, LDHs properties and its potential applications; (3) The method spent less time to carry out the synthesis and; (4) it did not need complicated solid-liquid separation processes.

## Graphical abstract

**Figure:**
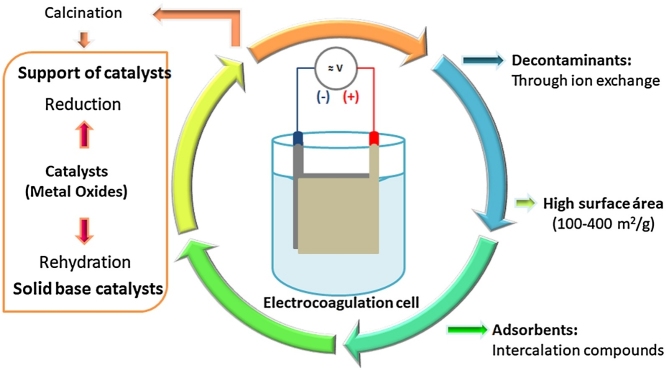

**Specifications Table**

**Table:** 

Subject area	*Chemical Engineering*
More specific subject area	Nanomaterial
Method name	Synthesis of Mg-Al layered double hydroxides by electrocoagulation
Name and reference of original method	Co-precipitation method is the most commonly used method to prepare hydrotalcites by addition of mixed solutions of divalent and trivalent metal salts in which all cations precipitate simultaneously.

## Method details

### Overview

Layered double hydroxides (LDHs) also know like hydrotalcites, have attracted enormous interest due to their many potential applications like anion exchangers, base catalysts, metal oxides precursors, PVC additives and drug delivery [1].

These LDHs materials can be describe using this general formula:

$$\left[{M\left(II\right)}_{1-x}{M\left(III\right)}_{x}{\left(OH\right)}_{2}\right]\left({A_{x/n}}^{n-}\right)∙mH_{2}O$$

Where $M\left(II\right)$ represents divalent cations such as ${Mg}^{2+}$, ${Fe}^{2+}$, ${Ni}^{2+}$, ${Cu}^{2+}$, ${Co}^{2+}$, ${Mn}^{2+}$, $.{Zn}^{2+}$ or ${Cd}^{2+}$, $M\left(III\right)$ denotes trivalent cations like ${Al}^{3+}$, ${Cr}^{3+}$, $.{Ga}^{3+}$ or ${Fe}^{3+}$, $A^{n-}$ is a $n^{-}$ valent exchangeable anion like ${{CO}_{3}}^{2-}$, ${{SO}_{4}}^{2-}$, ${Cl}^{-}$, ${{NO}_{3}}^{-}$, organic and inorganic anions, x varies between approximately 0.25 to 0.33 and m is the content of intercalated-water [2].

**Figure:**
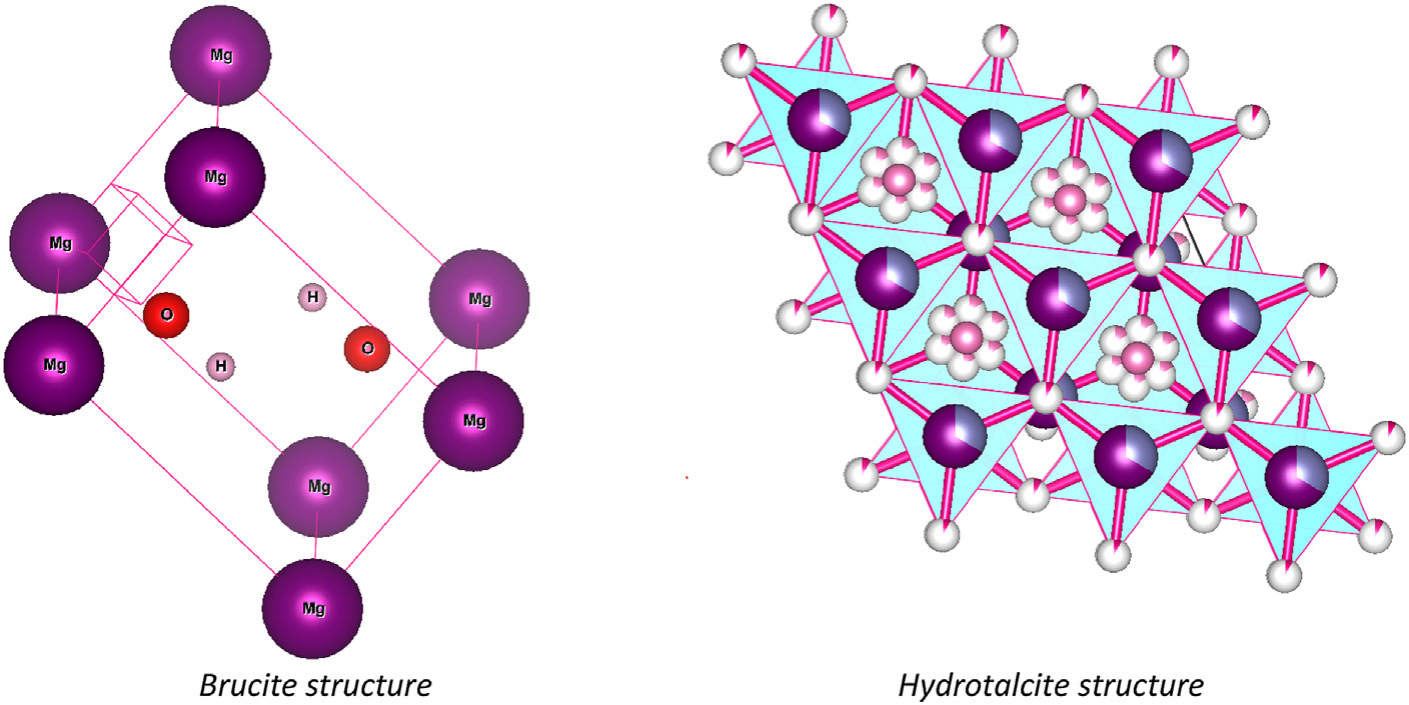

The major advantage of LDHs for their functional applications is their flexible composition, i.e., the ratio and nature of the divalent and trivalent metal cations and the varied interlayer structure through the substitution of many different anions in the interlayer of LDHs [3,4]. As a result, several types of hydrotalcites are used in various applications, depending on their composition, crystallinity, thermal stability and other physicochemical properties [[5], [6], [7]].

LDHs is generally synthesized under very strict experimental conditions in order to avoid chemical segregations and improve homogeneities, but the most common synthesis methods are co-precipitation at constant or variable pH [6]. The Urea decomposition method by hydrolyzing urea, using hexamethylene tetramine, creates an alkaline environment and well-crystallized LDHs. Another popular method is the Reconstruction from oxide generated by calcining LDHs, taking advantage of “memory effect” also allows the intercalation of any desired anion, such as biomedical or hazardous anions [[8], [9], [10]]. In general, there still exist some technological-economic problems involved in LDHs preparation. Although, the high maintenance costs and space requirements are drawbacks of the process [11,12].

This paper focuses in EC with the aim of identifying its potential effectiveness to synthesize LDHs. The employed strategy consists of the *in-situ* generation of coagulants, through electrochemical oxidation of two materials (anode and cathode), by switch polarity regularly during operation followed by solid-liquid separation.

Usually, Aluminum, Magnesium, Zinc or Iron plates are used as electrodes in the EC process. Mg^2+^ and Al^3+^ get hydrolyzed in water, dealing to the generation of different coagulants species:

• Insoluble hydroxides above which can enmesh the pollutants and/or
• Cationic or anionic hydroxocomplexes that destabilize electrostatic repulsion forces that exist in colloidal matter due to a charge neutralization mechanism or due to bound formation between particles.

In addition, other processes occur inside the electrochemical cell: close to the anode, oxygen generation due to water oxidation deals to the formation of H^+^ ions that, because of their charge, are attracted to the cathode. In the cathode, water reduction to hydrogen deals with the generation of hydroxyl ions OH^−^ that are attracted to the anode.

The EC method is interesting because no requires pH modifiers, neither chemical additives, demands less time for the materials crystallize into LDHs, It generates minimal sludge and requires regular replacement of sacrificial anode. During EC, it was demonstrated the successful anion adsorption in samples, at the same time, this novel method avoids the problem of recontamination caused by conventional techniques [13].

In this work, HDLs with high BET surface area have been synthesized by electrocoagulation from Aluminum and AZ31 magnesium alloys electrodes where Sodium Chlorine was used as electrolyte. The XRD and FTIR spectrum revealed peaks and bands characteristic of LDHs. The average crystallite size was calculated from background subtracted X-ray diffraction (XRD) patterns, by using Scherrer formula [14]. Indigo carmine solution also was investigated as electrolyte to determine if there was any change in the final product. While in a Sodium Chloride solution the EC produces a good quality LDHs of reasonable purity, Brucite is produced in the presence of Indigo Carmine. Besides, the charge density ratio $\left(M^{2+}/M^{3+}\right)$ could be controllable by varying the electrodes electro-dissolution time, throw the polarity control and aging time. Furthermore this advantageous properties, LDHs synthesized constitute an interesting alternative to environmental remediation technologies [15].

## Experimental

### Material and synthesis of LDHs

Electrocoagulation experiments were conducted in a batch mode, in a 1 L glass reactor using parallel electrodes plates with 0.9 L of solution prepared using reagents from AR grade. The electrocoagulation unit consisted in two Aluminum and AZ31 magnesium plates, as electrodes with an immersed area of 46.6 cm^2^ each one of them, a separation distance of 5 mm; a magnetic stirrer was used for mixing at 100 rpm. Electrodes were connected to a DC power supply and a manual polarity inverter unit, applying a current intensity of 5 mA at room temperature (24–26 °C).

**Fig. 1:**
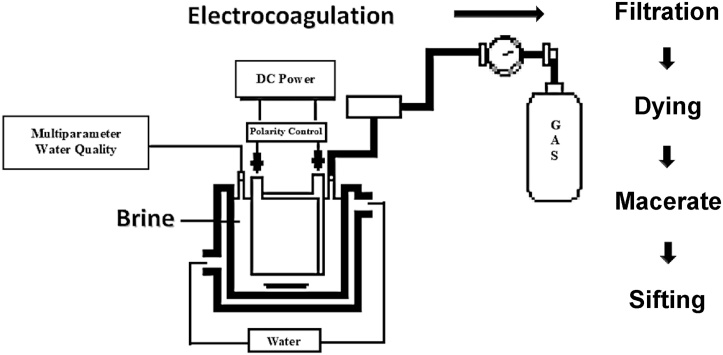
Description of the electrocoagulation cell and the process.

Experiments design in presented in Table 1 and the arrangement is presented in Fig. 1. Before testing, electrodes were subjected to dry abrasion with emery paper No. 600 and then with abrasive paper No. 1000. Afterward, the electrodes were rinsed with distilled water for about 5 min to remove metal traces.

**Table 1:** Experiments design.

Electrodes	Alternating polarization time (min)	Electrolyte-pollutant	Concentration (ppm)
AZ31-Al	8–5	NaCl-Indigo carmine	200–1800
AZ31-Al	8–5	NaCl	5000

The $\left(M^{2+}/M^{3+}\right)$ ratio and the operating time to get at specific electrical charge were calculated based on Faraday’s law, Eqs. (1) and (2), assuming that in a specific time only electrodissolution occurs at the anode.

(1) $$\left[Al\right]=\frac{I\times t\times M}{3\times F\times V}=\frac{3600\times Q\times M}{3\times F}$$

(2) $$\left[Mg\right]=\frac{I\times t\times M}{2\times F\times V}=\frac{3600\times Q\times M}{2\times F}$$

Where t is time (s), $\left[Al\right]$ is the amount of total Al generated (g L^−1^), $\left[Mg\right]$ is the amount of total Mg generated (g L^−1^), M is the molecular weight of Al ($M=26.98\;g\;{mol}^{-1}$) and Mg ($M=24.305\;g\;{mol}^{-1}$) and F is the Faraday constant ($F=96.485\;C\;{mol}^{-1}$), V is the initial volume in mL and I is the intensity in amperes.

Subsequently, the solution was quickly filtrated and dried in an oven at 105 °C for 2 h, and then macerated, and referred for XRD and FTIR analyses.

The chlorine concentration into the solution was calculated stoichiometrically before and after the electrocoagulation process in order to know the chlorine content adsorbed on the material. Atomic absorption was carried out in spectrophotometer Shimadzu model AA-7000 through measures of unreacted silver concentration after separation of the silver chloride formed by addition of silver nitrate in a liquid sample aliquot [16].

### Characterization

Synthesized LDHs were then characterized by X-ray diffraction (XRD), surface area analyses (BET) and Fourier-transformed infrared (FTIR). X-ray diffraction (XRD) pattern were recorder using a X’pert PRO – PANalytical diffractometer under the following conditions: 45 kV, 40 mA, monochromatic CuKα radiation $\left(\lambda =0.1542\;nm\right)$ in the 2 θ range of 4°–90°. The FTIR spectra were recorder with a JASCO FT/IR-4100 in a range between 500–4000 cm^−1^;samples were prepared by mixing the powdered solids with KBr. Surface area and pore analysis were performed using the Brunauer-Emmet-Teller (BET) approach on a Micrometrics ASAP 2020 adsorption analyzer. Zeta potential was measured using a Zeta-Meter 4.0 (Malvern Instruments, Malvern, UK) at 25 °C in clear disposable zeta cells, the pH and conductivity was measured using a Thermo Scientific Orion multiparameter (Star A329).

## Results and discussion

### Powder X-ray diffraction

**Fig. 2:**
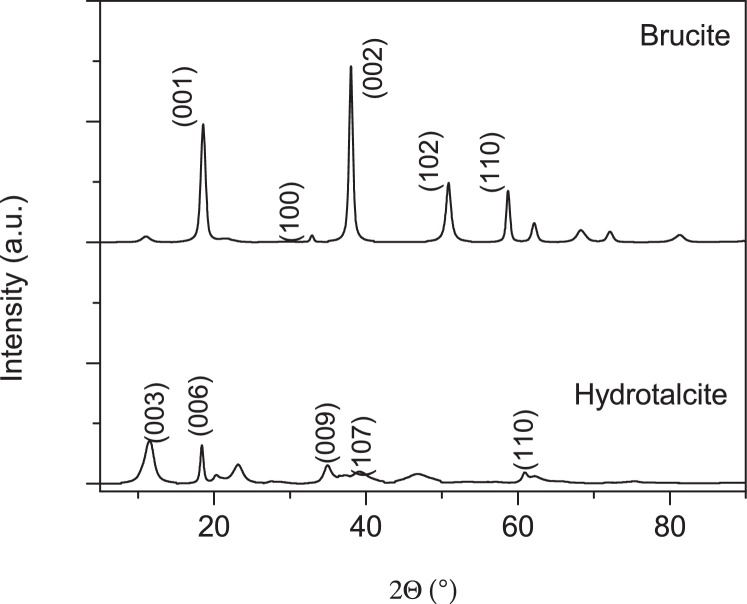
Comparison of XRD patterns of the synthesized products in the EC process.

Phase purity and crystallinity of the sludge were identified through XRD analysis. XRD patterns of product synthesized are shown in Fig. 2. Major diffraction peaks of LDHs were detected at 2θ angles of 11.827°, 23.373°, 34.076°, 36.147° and 62.097°, which are assigned to the crystal planes of (003), (006), (009), (107) and (110), respectively. Achieved peaks were similar to standard patterns of JCPDS file No: 98-000-6183, which stated the crystallographic system of $R\bar{3}m$ structure of LDHs. Diffraction peaks of Brucite matched with a standard pattern (JPCDS No. 98-003-4961).

In addition, Table 2 shows the phases and structural parameters obtained by X-ray diffraction. The total mass percentage observed for “AZ31-Al: Brucite” sample was 89.96% of Brucite and 10.03% of Disodium Carb; from “AZ31-Al: Hydrotalcite” sample was 72% of Hydrotalcite and 28% of Gibbsite. The presence of Gibbsite in the Mg-Al-LDH sample could be avoid using high temperature during EC process, however, more research are required after the synthesis of Mg-Al-LDH materials could be completely understood. LDHs crystallite size in perpendicular and parallel directions were 65.850 nm and 267.035 nm respectively; otherwise, Gibbsite crystallite size were 17.192 nm and 271.823 nm respectively, in same directions.

**Table 2:** Phases, lattice parameters and cell volume for the samples.

Sample	Lattice parameters (Å)				Cell volume (10^6^pm^3^)				
	Hidrotalcite	Gibbsite	Brucite	Carbon	Hidrotalcite	Gibbsite	Brucite	Carbon	χ²
AZ31-A:l Brucite			a = 3.14	a = 13.52					2.12
			b = 3.14	b = 13.52			40.96	2472	
			c = 4.77	c = 13.52					
AZ31-Al: Hydrotalcite	a = 3.05	a=4.95			185.076	420.74			1.1
	b = 3.05	b = 9.67							
	c = 22.97	c = 8.79							

### Specific surface area

Table 3, shows the specific surface area, the pore volume and pore size of the synthesized products. Typical values of the specific surface area measured by the BET technique range from 24.6 to 69 m^2^/g and are related with the efficiency of the synthesis process [17]. For synthesized hydrotalcites, BET surface area (Table 3) were larger than reported on literature. Thus, to explain this result the effect of the mixer velocity should be considered as key parameter in the bubble production, that makes easier the breaking and exfoliation of hydrotalcite layers during EC process.

**Table 3:** Textural properties of the synthesized products.

Sample	S_BET_ (m^2^/g)	Pore volume (cm^3^/g)	Average pore size (Å)
AZ31-Al: Brucite	220.008	0.354	64.414
AZ31-Al: Hydrotalcite	206.375	0.359	69.659

### Zeta potential, pH and conductivity

The surface charge of the LDHs could have an important implication on their mobility and suspension stability in water. This value decides the level of the electrostatic repulsion between particles. The ZP of the particles in suspension is positive during the process and moderate stability at 39 mV and 30 mV values for the “AZ31-Al: Brucite” and “AZ31-Al: Hydrotalcite” experiments respectively. pHs were also measured and varies from 6.5 to 10.5 for “AZ31-Al:Brucite” and from 7.5 to 11.5 for “AZ31-Al: Hydrotalcites” the pH raises as a consecuence of OH^−^ production during electrocoagulation, it is shown in the high pH buffering capacity of LDHs [18]. On the other hand, the average conductivity were 3.4 mS/cm and 8.6 mS/cm. The chloride anion significantly affects the zeta potential of the LDH particles.

### Fourier transform-infrared (FTIR) spectroscopy

**Fig. 3:**
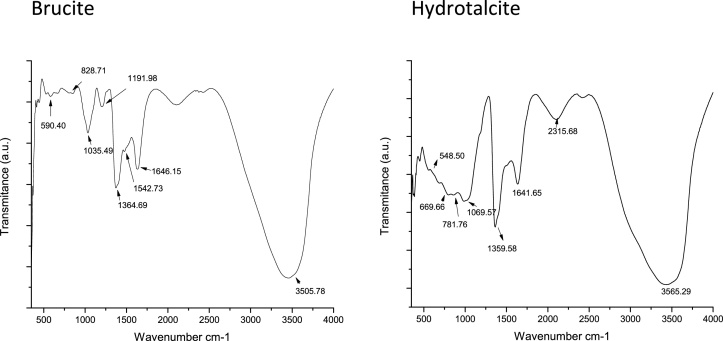
FTIR of the synthesized products in the EC process.

Fig. 3, illustrates FTIRs of the samples. From “AZ31-Al: Hydrotalcite” sample, characteristic peaks observed at 574 cm^−1^ and 3636 cm^−1^ were assigned to the stretching of metal-oxygen due to the Al—O and O—H group, respectively. Strongest peaks at 3565.29 cm^−1^ and 3500.61 cm^−1^ corresponded to M—OH modes. Peaks at 1641.65 cm^−1^ and 1359.58 cm^−1^ were assigned to the water deformation mode, while the peak around 1069.57 cm^−1^ was assigned to carbonate mode. Bands around 548.50 cm^−1^ and 505.64 cm^−1^ could be assigned to Al−OH and Mg−OH translation modes.

Peaks observed for “AZ31-Al: Brucite” sample reports the presence of Brucite, the peak at 3505.78 cm^−1^ was assigned to M—OH mode. The peak at 1646.15 cm^−1^ shows the presence of inter-lamellar water. Peaks at 590.40 cm^−1^ and 517.61 cm^−1^ were assigned to Al—OH and Mg—OH.

### Properties and applications

For LDHs synthesized, physical properties such as: surface area, pore size and pore volume observed are extremely important factors to their description and define their applications in many different areas. Besides, based on the results, in solution, these composites presents a positive zeta potential of ≈30 mV resulting from the combination of charges from the edges and the fundamental surfaces, which is important for systems with colloidal characteristics, although this value limits their exchange/adsorption applications.

Anion uptake characteristic properties of LDHs synthesized were investigated through the measure of chlorine concentration into the solution. It was found an overall chloride in-situ removal of 63% corresponding to a reaction-process time of 3.15 h. These results are of great interest for environment remediation technologies.

### Material consumption and electrical operating costs

Cost of LDHs were calculated considering the reported by Secula et al. [19] using the following Eqs. (3) and (4):

(3) EOC=EEC+EMC

Where EOC refers to electrical operating cost (USD/Kg) of LDHs synthesized; ECC the energy consumption (USD/Kg); and, EMC the electrode material cost (USD/Kg).

(4) $$EOC=UED\times EEP+UEMD_{Al}\times EMP+UEMD_{AZ31}\times EMP$$

In Eq. (4), UED represents the unit energy demand (KWh/Kg); EEP the electrical energy price (USD/KWh); UEMD the unit electrode material demand (which is 1/3 for Al and 2/3 for Mg); and, EMP the electrode material price (USD/Kg). Considering the current intensity was kept constant and cell voltage varied during electrocoagulation process, by the Eq. (5):

(5) $$UED=I\int_{0}^{t}U∙dt$$

Where I is the current intensity, (A); U the cell voltage, (V), t the time, (h). EMP were estimated from international suppliers as 2.7 USD/Kg for Aluminum and 25 USD/Kg for AZ31 magnesium alloys. EEP were determined to be 0.315 USD/KWh and the UED was of 0.5880 KWh/kg. EMP related to AZ31 presents a value of 25 USD/Kg, this value can be optimized using high purity magnesium as electrode, because the price of this material is 10 times lower than the one for the alloy.

## Conclusions

In summary, electrocoagulation process using aluminum, AZ31 magnesium electrodes and Sodium Chlorine offers a method for the synthesis of Hydrotalcite-like layered double hydroxides, in spite of the same process leads to Brucite formation when a combination of Indigo Carmine and Sodium Chloride was used. As shown in the X-ray results, in the composites synthesized can exists Gibbsite, a common impurity present in conventional LDHs synthesis process. So, pollutants composition in water affects the generation and the type of LDHs produced.

Based on the results, EMP related to AZ31 presents a value of 25 USD/Kg that correspond to a high material cost but this value can be optimized using high purity magnesium as electrode in order make possible their applicability at industrial scale.

In conclusion, results demonstrated that Hydrotalcite-like layered double hydroxides prepared from electrocoagulation method using synthetic water under laboratory scale conditions, have a greater surface area, indicating a great potential for the remediation technologies. Finally, this synthesis could be useful for several future applications because it avoids the use of pH modifiers and the electrodes can be replace in order to synthesize different LDHs composites.
